# Anti-Inflammatory and Immunomodulatory Effects of Intravenous Lidocaine in Surgery: A Narrative Review

**DOI:** 10.3390/jcm14113883

**Published:** 2025-05-31

**Authors:** Ana Fernández-Martínez, Joseba González García, Amanda López-Picado

**Affiliations:** 1 Anaesthesiology Department, Hospital Universitario San Pedro de Logroño, 26006 Logroño, Spain; dra.fernandez.anesthesia@gmail.com; 2 Anaesthesiology Department, Hospital Universitario de Basurto, 48013 Bilbao, Spain; josebagonzalezgarcia@outlook.com; 3 Health Faculty, Universidad Internacional de La Rioja, 26006 Logroño, Spain

**Keywords:** intravenous lidocaine, anti-inflammatory effect, immunomodulation, anaesthesia and immune system, perioperative medicine

## Abstract

Lidocaine, a widely used local anaesthetic, has been shown to possess anti-inflammatory and immunomodulatory properties with applications in surgery. During a surgical procedure, inflammation is a natural response of the body, triggered by the release of inflammatory mediators and the activation of the immune system. However, an excessive response can lead to serious postoperative complications. Lidocaine modulates inflammation through mechanisms beyond its anaesthetic action. It reduces the activation of neutrophils and macrophages, decreases the release of pro-inflammatory cytokines and prostaglandins, and preserves endothelial integrity, helping to control excessive inflammatory responses. Additionally, its perioperative use has shown benefits such as reduced postoperative pain, lower opioid consumption, and faster intestinal recovery. Furthermore, studies have suggested that lidocaine may have an anti-metastatic effect by inhibiting the migration of tumour cells and the activation of inflammatory pathways related to cancer spread. Although its use in surgery is promising, further research is needed to determine optimal dosages and its long-term clinical impact.

## 1. Introduction

Surgical procedures entail tissue injury and trauma, and exposure to commensal microorganisms, resulting in a local inflammatory response [1]. This response is characterised by increased vascular permeability and blood flow, alongside the release of inflammatory mediators [2]. In addition, patients are subjected to invasive techniques such as venous cannulation or orotracheal intubation, which can trigger inflammatory reactions at sites distant from the surgical field. The release of both pro-inflammatory and anti-inflammatory mediators, and the ensuing immune response, will ultimately determine the outcome of this process. However, it is not possible to predict which patients will develop inflammation-related complications, nor are there specific therapeutic strategies capable of preventing their occurrence.

The degree of inflammation associated with each surgical procedure varies depending on the extent of physical trauma, microbial exposure, and patient-specific factors such as age and comorbidities [1]. Moreover, factors such as ischaemia–reperfusion injury, tissue hypoxia, transfusion of blood products, and mechanical ventilation may further exacerbate the inflammatory response [3]. Postoperatively, an excessive innate immune response or an insufficient adaptive response may lead to significant morbidity and mortality [1]. It is therefore essential to develop and implement strategies that modulate the inflammatory response associated with surgery. Current approaches include minimising surgical trauma, reducing operative time, and administering drugs with anti-inflammatory and immunomodulatory properties, such as lidocaine [3].

Although the anti-inflammatory and antitumour properties of lidocaine have been extensively documented in numerous in vitro and in vivo studies, the underlying mechanisms remain incompletely understood. When administered intravenously in the perioperative setting, lidocaine has been shown to reduce opioid requirements, postoperative pain, paralytic ileus, and hospital length of stay. However, its role in modulating the inflammatory and immune response to surgery remains unclear. Existing evidence is limited by variability in surgical procedures, dosing regimens, and duration of treatment. Consequently, there is a significant knowledge gap concerning the mechanisms and potential clinical benefits of its anti-inflammatory and immunomodulatory actions. Addressing this gap is crucial, as it may contribute to the reduction in postoperative complications related to local and systemic inflammation—such as surgical site infections, sepsis, anastomotic dehiscence, cardiorespiratory complications, and even tumour recurrence or metastasis in oncological patients [3].

To guide the reader through the main themes of this narrative review, a summary of the key points is provided in Table 1.

## 2. Methodology

A narrative review was conducted instead of a systematic review due to the complexity and breadth of the topic. The anti-inflammatory and immunomodulatory effects of lidocaine have been investigated across a wide range of contexts—including animal models, in vitro studies, clinical trials, and observational studies in various clinical settings—resulting in a highly heterogeneous body of literature in terms of objectives, study design, and methodological quality. This heterogeneity poses significant challenges to the application of the strict selection criteria required for a systematic review.

The narrative review approach enables a broader and more critical integration of the available findings, highlighting pathophysiological mechanisms, emerging clinical hypotheses, and potential therapeutic implications. This type of review is particularly valuable in areas where the current evidence is insufficiently uniform to support meta-analysis, and it aims to provide a comprehensive synthesis that may inform future research and support clinical decision-making.

A literature search was conducted using the Medline and PubMed databases. The search strategy included the following terms: (anti-inflammatory effect) AND (lidocaine) and (systemic inflammation) AND (lidocaine). The following filters were applied to refine the search: review, systematic review, clinical trial, and meta-analysis, with a publication window limited to the last 10 years. In addition, studies referenced in the bibliographies of key selected articles were included due to their relevance and significance.

## 3. Review of the Evidence

### 3.1. Inflammation and Surgery

Surgical procedures trigger a complex cascade of inflammatory events mediated by the release of damage-associated molecular patterns (DAMPs), which are endogenous molecules acting as danger signals to the immune system. These signals are recognised by Toll-like receptors (TLRs) expressed on the surface of endothelial, somatic, and haematopoietic cells, leading to the activation of immune cells such as neutrophils and monocytes. This process promotes the expression of adhesion molecules, cellular recruitment, and the release of pro-inflammatory mediators, forming an initial inflammatory response aimed at restoring tissue homeostasis. The vascular endothelium, traditionally viewed as a passive barrier, plays an active role as a sensor and integrator of inflammatory signals. In response to pro-inflammatory cytokines, endothelial cells amplify the immune response via transcriptional activation, de novo protein synthesis, and the expression of adhesion molecules and tissue factors [4].

Although a moderate inflammatory response is essential for tissue repair and infection control, excessive immune activation may be harmful. Such responses have been associated with organ dysfunction (e.g., renal, pulmonary), postoperative infections, delirium, and pain, all of which contribute to increased morbidity and prolonged hospital stay. Moreover, certain cytokines—such as IL-1RA, IL-4, and IL-10—foster immunosuppression by inhibiting effector cell activity, thereby increasing susceptibility to infection and facilitating tumour persistence or dissemination during the postoperative period [5].

Among the various factors that intensify the inflammatory response, surgical duration is one of the most influential, as peak cytokine release typically occurs at the end of the procedure and is independent of preoperative levels [6]. Additionally, perioperative factors such as mechanical ventilation with high tidal volumes (10–12 mL/kg) can induce the release of inflammatory mediators such as TNF-α and IL-8 [7]. An excessive inflammatory response may culminate in an uncontrolled cytokine surge—commonly referred to as a “cytokine storm”—contributing to cell death and multiorgan damage, as observed in conditions such as ischaemia–reperfusion injury and SARS-CoV-2 infection [8].

Endothelial damage associated with systemic inflammation involves the generation of reactive oxygen species (ROS), degradation of the glycocalyx, and enhanced recruitment of immune cells, perpetuating a vicious cycle that sustains the inflammatory state. Immune activation may also be driven by pathogen-associated molecular patterns (PAMPs) in cases of surgical infection. Due to structural similarities between PAMPs and DAMPs, the immune system may struggle to distinguish sterile surgical injury from active infection [4].

Even minimally invasive procedures elicit a local inflammatory response. The skin acts as a reservoir of chemokines, and its manipulation leads to the simultaneous release of pro- and anti-inflammatory cytokines. Experimental mouse models have shown that the nature of the haemorrhage and the type of tissue injury are key determinants of the resulting immune profile [4,9]. A paradigmatic example is ischaemia–reperfusion injury, as occurs during vascular clamping or organ transplantation, where endothelial oedema, ROS generation, glycocalyx disruption, neutrophil infiltration, and eventual apoptosis and necrosis of renal tubular cells further amplify the inflammatory response [4].

Among the most relevant DAMPs released in the surgical setting are the S100 proteins and high mobility group box 1 (HMGB1) protein. These are recognised by a variety of pattern recognition receptors (PRRs), including mannose-binding lectin (MBL), TLRs, and receptors for advanced glycation end-products (RAGEs), and their elevated plasma concentrations have been correlated with the extent of surgery-induced inflammation [4].

### 3.2. Immunomodulatory and Antimetastatic Effects of Lidocaine

Surgery remains a cornerstone in cancer treatment; however, paradoxically, it may also promote biological processes that contribute to tumour recurrence and metastatic spread. In a study involving patients undergoing mastectomy for breast cancer, two peaks of disease recurrence were identified: an early peak at 18 months and a later one at 60 months. While the latter likely reflects the natural course of oncological progression, the early recurrence has been associated with the intraoperative release of viable tumour cells [10].

During surgical intervention, the release of inflammatory mediators such as prostaglandins, cytokines, and catecholamines can induce transient immune dysfunction, particularly affecting Natural Killer (NK) cells, which play a central role in eliminating circulating tumour cells. In addition, increased levels of pro-angiogenic and tumour-promoting factors—such as vascular endothelial growth factor (VEGF) and transforming growth factor-beta (TGF-β)—have been reported. Furthermore, the manipulation and resection of the primary tumour may facilitate the dissemination of malignant cells and the establishment of distant metastatic foci [11].

In this context, amide-type local anaesthetics such as lidocaine and ropivacaine have been proposed to exert immunoprotective and antimetastatic effects. Several studies have demonstrated their ability to modulate endothelial inflammatory responses, preserve vascular integrity, and thereby reduce tumour cell extravasation. These agents may also inhibit neutrophil migration—an important mechanism given that tumour cells can exploit neutrophil adhesion, mediated by intercellular adhesion molecule-1 (ICAM-1), to traverse the endothelium more easily [11].

At the molecular level, there is significant overlap between inflammatory pathways and those involved in tumour progression. A key element is the Src tyrosine kinase, whose activation by cytokines such as TNF-α compromises endothelial barrier integrity, facilitating tumour cell migration, invasion, and extravasation [11]. Lidocaine and ropivacaine have been shown to inhibit Src activation and ICAM-1 phosphorylation in tumour cells. An in vitro study demonstrated that incubating lung cancer cells with TNF-α in the presence of these anaesthetics (1 nM–100 μM) significantly reduced cell migration, suggesting a mechanism of action independent of their sodium channel-blocking effects, traditionally associated with anaesthesia [12].

Beyond their impact on the endothelium and immune response, lidocaine has shown direct cytotoxic effects on various tumour cell lines. It has been reported to induce apoptosis and inhibit proliferation in models of breast cancer [13], pulmonary adenocarcinoma [14], thyroid cancer [15], and hepatocellular carcinoma [16]. In murine models, lidocaine administration has also reduced the growth of subcutaneous human hepatocellular carcinoma tumours, exerting effects comparable to cisplatin and enhancing tumour sensitivity to it, through apoptosis induction and G0–G1 phase cell cycle arrest [17]. These findings support the hypothesis that lidocaine could serve as a therapeutic adjunct in oncological surgery.

Although clinical evidence remains inconclusive, several studies have explored the impact of anaesthetic technique and local anaesthetic use on cancer outcomes. In breast surgery, for instance, regional anaesthesia using local anaesthetics has been proposed to offer advantages over opioid-based approaches. One study in oestrogen receptor-negative breast cancer patients reported increased tumour cell apoptosis in those receiving propofol and paravertebral block compared to those treated with sevoflurane and opioids [18].

### 3.3. Anti-Inflammatory Effect of Lidocaine

Intravenous administration of lidocaine during the intraoperative period has been shown to reduce systemic inflammatory responses, with effects that persist beyond its half-life and metabolism to inactive forms [5]. At the usual doses used in the perioperative setting, the plasma concentrations reached exert minimal blockade of sodium channels, suggesting that its anti-inflammatory and immunomodulatory effects stem from additional molecular mechanisms that are yet to be fully defined [3].

Several studies have demonstrated that lidocaine modulates leukocyte function, suppressing their activation, migration, and adhesion, and the release of inflammatory mediators, such as superoxide anions, prostaglandins, interleukins, gamma interferon (IFN-γ), tumour necrosis factor alpha (TNF-α), and beta (TNF-β) [5]. Some of these findings are not recent; as early as the 1980s, studies observed that lidocaine inhibited the activation and adhesion of polymorphonuclear cells in animal models [19,20]. At the cellular level, its action appears to be mediated by interference with membrane ion transporters and alteration of intracellular pH, which contributes to the inhibition of cytokine release [2].

A recent meta-analysis confirmed that intravenous administration of lidocaine in elective surgery reduces levels of IL-6, IL-1RA, CRP, and TNF-α, especially in open surgery [5]. Furthermore, it has been reported that doses of 1.5 mg/kg/h reduce concentrations of pro-inflammatory cytokines and alter the expression of microRNA (miRNA), non-coding molecules involved in the post-transcriptional regulation of processes such as apoptosis and inflammation [21].

From a biochemical perspective, it has been demonstrated that lidocaine inhibits the biosynthesis of prostaglandins and thromboxane B2, both derived from arachidonic acid metabolism through the action of phospholipase A2 and cyclooxygenase [22,23]. This action could explain, at least in part, its beneficial effect on platelet aggregation and thrombotic risk [24,25]. The inhibition of prostaglandins has also been proposed as a mechanism responsible for the analgesic and anti-inflammatory effects observed in burn patients treated with lidocaine [26,27], and it has been described that lidocaine modulates the activity of phospholipase A2 in a dose-dependent manner, inhibiting its activity at high concentrations and increasing it at low concentrations [28].

In experimental models of lung injury, lidocaine has demonstrated anti-inflammatory and cytoprotective effects mediated by modulation of the MAPK signalling pathway, which is involved in extracellular and intracellular signal transduction [29]. In pigs subjected to one-lung ventilation and pulmonary resection surgery, a reduction in the release of inflammatory mediators, cellular apoptosis, and pulmonary oedema was observed 24 h postoperatively, without compromising haemodynamics or oxygenation [30].

Furthermore, lidocaine appears to interfere with the activation of hypoxia-inducible factor 1-alpha (HIF-1α), which regulates genes involved in cellular responses to hypoxia, angiogenesis, and tumour dissemination [31].

Via the intrathecal route, lidocaine has also demonstrated anti-inflammatory effects. In murine models, it increased the pain threshold and inhibited microglial activation, which was associated with a higher expression of SOCS3, a protein that regulates cytokine signalling [32]. This effect seems to contribute to the potentiation of opioid-induced analgesia and the reduction in opioid tolerance by upregulating the same SOCS3 molecules [33]. In another study involving rats, systemic administration of lidocaine prior to exposure to kainic acid—a neurotoxic glutamate analogue—reduced neuronal death, microglial activation, and the expression of IL-1β, IL-6, and TNF-α [34]. These findings may correlate with the clinical results from a trial in which patients treated with intraoperative lidocaine experienced less postoperative cognitive decline and better scores on the Mini-Mental State Examination [35].

**Figure 1:**
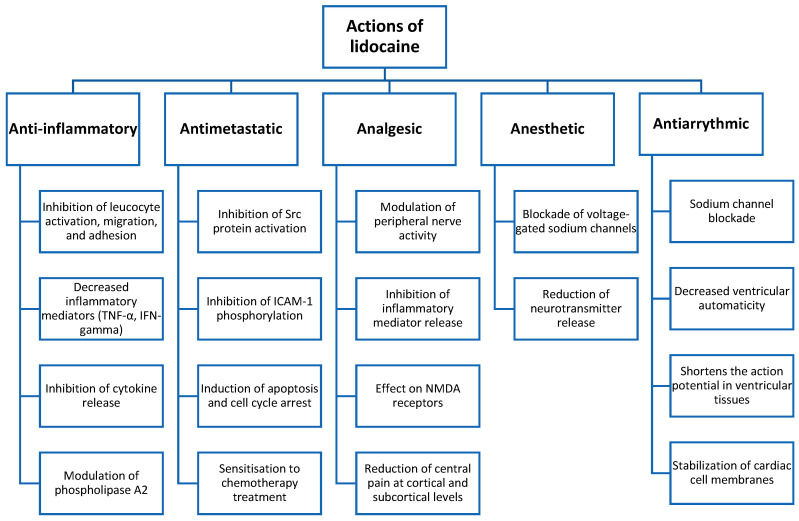
Diagram of the actions of systemic lidocaine.

The main actions of intravenous lidocaine are summarised in Figure 1.

### 3.4. Pharmacokinetics, Dosing, and Toxicity

The pharmacokinetic properties of lidocaine have been well characterised in the context of its use as an antiarrhythmic. Following intravenous administration, it is rapidly distributed (distribution half-life of 5 to 8 min) following a multi-compartment model, with an initial phase towards highly perfused organs and a second phase towards peripheral tissues [36]. Its hepatic metabolism, mediated by cytochrome P450, and its high hepatic extraction ratio (70%) make hepatic blood flow a critical determinant of its clearance. The half-life in healthy adults ranges from 80 to 110 min. The active metabolites, monoethylglycinexylidide and glycinexylidide, have a lower potency than the original molecule; however, the former retains antiarrhythmic activity and a potential convulsant effect [37]. Lidocaine binds to α1-acid glycoprotein in an inverse proportion to its plasma concentration, complicating the definition of a precise toxic threshold, as only the free fraction is pharmacologically active. Clinically, the administration of an initial bolus is essential to rapidly reach therapeutic plasma concentrations; without this, continuous infusion may take hours to achieve effective levels [36].

Perioperative intravenous administration of lidocaine is typically performed with an initial bolus of 1 mg/kg, followed by continuous infusion at a rate of 0.5 to 3 mg/kg/h, with 2 mg/kg/h being the most studied and commonly used in clinical practice. This regimen achieves plasma concentrations of approximately 2 µg/mL, sufficient to modulate the sympathetic response to surgical stress, reduce pain, and decrease the requirements for inhalational agents and opioids [38]. If intravenous infusion is maintained beyond 24 h, a decrease in the clearance of lidocaine may occur due to hepatic enzyme saturation and competition with its active metabolites for binding sites. Therefore, it is recommended to adjust the dosage based on total body weight and consider reducing the infusion rate after 24 h to avoid systemic toxicity [36].

Lidocaine toxicity is due to its blocking action on sodium channels and, to a lesser extent, potassium channels, affecting both the central nervous system and the cardiovascular system. The initial signs of toxicity are typically neurological and include a metallic taste, dizziness, tinnitus, and paraesthesia. In more severe cases, seizures, arrhythmias, and cardiac arrest may occur [36]. Although its cardiotoxic profile is more favourable than that of bupivacaine, at concentrations greater than 10 µg/mL, it may prolong the PR interval, widen the QRS complex, and induce bradycardia and hypotension. Toxicity manifestations in the central nervous system are generally observed from plasma concentrations of 5 µg/mL, with seizures becoming more frequent when exceeding 15 µg/mL, originating in the amygdala and rapidly generalising. No increased risk of lidocaine-induced seizures has been observed in patients with a history of partial seizures compared to the general population [39].

It is essential to individualise the dose of lidocaine in special populations with altered metabolism or distribution, as this may increase the risk of toxicity. In patients with advanced hepatic cirrhosis (Child C), the dose should be reduced by half due to reduced hepatic blood flow. In cases of severe renal insufficiency, it should be considered that the elimination half-life may double. In elderly individuals, the volume of distribution is reduced; therefore, the loading dose should be adjusted to body weight, and the intravenous infusion rate should be reduced by 35%. In obese patients, due to the increased volume of distribution associated with body weight, it is suggested that the loading dose be calculated based on actual body weight, with continuous infusion adjusted according to ideal body weight [36].

### 3.5. Abdominal Surgery

The pain associated with abdominal surgery results from a combination of somatic (skin incision) and visceral (mechanical traction, inflammation, ischaemia, and nerve injury) stimuli, which justifies the benefit and effectiveness of multimodal analgesia in this type of surgery [3]. In this context, perioperative intravenous administration of lidocaine has demonstrated beneficial effects in multiple studies, mainly attributed to its anti-inflammatory action, although questions remain regarding the optimal dose and the ideal duration of treatment [40].

In patients undergoing elective laparoscopic cholecystectomy, lidocaine administered both intraperitoneally and intravenously has been shown to significantly reduce postoperative pain and opioid consumption [40]. In fact, patients treated with intravenous lidocaine had better pain control compared to those receiving diclofenac and paracetamol [41]. Furthermore, its combination with dexmedetomidine and propofol provides an effective alternative for pain management, especially in patients at a high risk of postoperative nausea and vomiting [42]. However, some studies have not demonstrated significant clinical benefits, despite observing a reduction in inflammatory markers such as IL-1, IL-6, TNF-α, and IFN-gamma. In these cases, no differences were found in opioid consumption, the occurrence of paralytic ileus, pain intensity, or hospital stay [43].

In colorectal and major abdominal surgery, intravenous lidocaine has shown efficacy comparable to epidural analgesia with local anaesthetics in terms of pain control, reduced opioid consumption, prevention of paralytic ileus, and decreased hospital stay [44,45,46]. It has also demonstrated superiority over transversalis fascia block, possibly due to its anti-inflammatory effect and its ability to reduce postoperative nociceptive hypersensitivity [47].

In the context of bariatric surgery, intravenous lidocaine administered perioperatively has been effective in reducing opioid consumption during the first 24 h [48], improving analgesia, reducing the incidence of postoperative ileus, and decreasing nausea and vomiting, partly due to reduced opioid use [49,50,51].

### 3.6. Genitourinary Surgery

Intravenous lidocaine administered during the perioperative period has shown beneficial effects in the context of genitourinary surgery. In patients undergoing radical retropubic prostatectomy, its use has been associated with a significant reduction in postoperative pain, opioid consumption, and hospital stay, as well as faster recovery of intestinal function [52]. In open renal surgery, a decrease in intraoperative requirements for isoflurane and remifentanil was also observed [53]. However, these effects were not found in laparoscopic renal surgery, with no significant benefits identified [54].

### 3.7. Gynaecological and Obstetric Surgery

In gynaecological surgery, the effects of intravenous lidocaine appear to depend on the type of surgical approach. In laparoscopic procedures, such as total hysterectomy, various clinical trials have shown improved pain control and reduced opioid consumption [55,56]. However, these effects have not been consistently observed in open gynaecological surgery, suggesting that the beneficial effects of lidocaine may be conditioned by the lesser tissue trauma and inflammatory response associated with minimally invasive surgery.

In obstetric practice, a randomised clinical trial evaluated the impact of intravenous lidocaine during elective caesarean sections, observing a reduction in the maternal stress response, as measured by plasma cortisol levels, and a lower elevation in heart rate and blood pressure following the administration of a 1.5 mg/kg bolus, followed by a continuous infusion at the same dose for one hour. No neonatal adverse effects were observed, as Apgar scores and acid–base balance values were similar between the intervention and control groups. Despite these promising results, current evidence remains limited, and routine use in this population is not recommended without further studies supporting its safety and efficacy [57].

### 3.8. Breast Surgery

In the context of breast surgery, current evidence indicates that perioperative intravenous administration of lidocaine does not confer significant short-term benefits in patients undergoing total mastectomy. Placebo-controlled studies have not demonstrated relevant differences in opioid consumption, postoperative pain control, incidence of nausea and vomiting, or length of hospital stay [58]. However, a significant reduction in chronic postmastectomy pain has been observed at 3 and 6 months postoperatively in patients who received lidocaine, suggesting a potential long-term benefit [59,60].

### 3.9. Cardiac Surgery

In cardiac surgery, a clinical trial evaluated the effect of lidocaine on intraoperative cerebral inflammation in patients undergoing cardiopulmonary bypass. The results showed a reduction in the transcranial activation of platelet–monocyte conjugates following aortic unclamping, suggesting modulation of the cerebral inflammatory response induced by extracorporeal circulation [61].

### 3.10. Thoracic Surgery

Perioperative intravenous lidocaine has shown efficacy in postoperative pain control and reduced morphine consumption in thoracic surgery, constituting a valid alternative for patients in whom thoracic epidural analgesia is contraindicated or not feasible [62]. These findings have also been reported in patients undergoing pulmonary resection surgery [63]. However, in the case of video-assisted thoracoscopic surgery (VATS), no clinically relevant benefits have been observed compared to placebo in terms of postoperative pain, incidence of nausea and vomiting, or duration of stay in the Post-Anaesthesia Care Unit [64].

### 3.11. Hip and Spine Surgery

In hip surgery, intravenous lidocaine has not demonstrated significant advantages compared to placebo [65]. Conversely, more promising results have been described in major spinal surgery, where its administration has been associated with a significant reduction in pain and a sustained improvement in quality of life at one and three months postoperatively [66]. This suggests a potential long-term benefit, similar to that observed in chronic postmastectomy pain [59,60].

## 4. Limitations

This narrative review has several limitations that should be considered when interpreting its findings. While the narrative approach allows for a broader exploration of the topic, it does not follow a predefined or standardised protocol, limiting the reproducibility of the search strategy and selection of included studies. Likewise, there is an inherent risk of author bias in the selection, organisation, and interpretation of the literature, which may have influenced the emphasis placed on certain aspects of the topic.

Furthermore, the methodological quality and risk of bias of the included studies were not formally or systematically assessed, potentially affecting the robustness of the evidence presented. The inclusion of highly heterogeneous studies—such as in vitro research, animal models, and clinical trials with varying designs and populations—makes direct comparison of the results challenging and limits the ability to draw conclusions applicable to routine clinical practice. While preclinical studies contribute valuable insight into underlying pathophysiological mechanisms, their findings are not always translatable to humans. Despite these limitations, this review offers an integrated and critical overview of the current knowledge on the effects of intravenous lidocaine on the inflammatory response and immune modulation in the surgical context, and may serve as a foundation for future systematic investigations.

## 5. Conclusions

Lidocaine has demonstrated anti-inflammatory, immunomodulatory, and antitumour properties in numerous in vitro and in vivo studies. Its perioperative intravenous administration has been shown to reduce postoperative pain, opioid consumption, paralytic ileus, and hospital stay across various surgical settings, with the most robust evidence emerging in abdominal surgery. Despite these promising findings, the variability in effects depending on surgical type, dosage, and patient characteristics suggests that further clinical studies are needed to more precisely define the optimal dosing regimens and duration of administration, and ultimately, to develop standardised protocols that maximise therapeutic benefits while minimising the risk of toxicity [5].

## Figures and Tables

**Table 1:** Summary of key points.

**Background**	Surgical procedures and associated invasive techniques trigger an inflammatory response, which, although necessary to some extent, can lead to serious postoperative complications if excessively activated. These complications may include sepsis, anastomotic dehiscence, and cardiopulmonary events, among others. Lidocaine has been shown in multiple in vitro and in vivo studies to modulate the immune system and attenuate the inflammatory response associated with surgical trauma.
**Methodology**	A literature search was conducted using PubMed databases. The search strategy included the following terms: (anti-inflammatory effect) AND (lidocaine) and (systemic inflammation) AND (lidocaine). The following filters were applied: reviews, systematic reviews, clinical trials, and meta-analyses published within the last 10 years. Relevant studies cited in the bibliographies of key selected articles were also included.
**Results**	Perioperative intravenous administration of lidocaine has been shown in multiple studies to reduce postoperative pain, opioid consumption, paralytic ileus, and length of hospital stay, particularly in abdominal surgery, where it has been most extensively studied. In spinal and breast surgery, lidocaine has demonstrated efficacy in reducing chronic postoperative pain for up to three months. Furthermore, it appears to reduce the intraoperative dissemination of tumour cells during oncological procedures and modulates the immune system by promoting tumour cell apoptosis and cell cycle arrest.
**Conclusions**	Intravenous lidocaine shows promising potential as an immunomodulatory agent in surgical settings. However, further research is needed to establish optimal dosing regimens, determine the appropriate duration of administration, and assess its clinical impact in both the short and long term.

## References

[1] Alazawi W., Pirmadjid N., Lahiri R., Bhattacharya S. (2016). Inflammatory and Immune Responses to Surgery and Their Clinical Impact. Ann. Surg..

[2] Karnina R., Arif S.K., Hatta M., Bukhari A. (2021). Molecular mechanisms of lidocaine. Ann. Med. Surg..

[3] Wang J., Bian Q., Chen X., Feng Y., Zhang L., Chen P. (2024). The mechanism of perioperative intravenous lidocaine in regulating the inflammatory response: A review. Medicine.

[4] Margraf A., Ludwig N., Zarbock A., Rossaint J. (2020). Systemic Inflammatory Response Syndrome After Surgery: Mechanisms and Protection. Anesth. Analg..

[5] Castro I., Carvalho P., Vale N., Monjardino T., Mourão J. (2023). Systemic Anti-Inflammatory Effects of Intravenous Lidocaine in Surgical Patients: A Systematic Review and Meta-Analysis. J. Clin. Med..

[6] Perioperative Cytokine Release During Coronary Artery Bypass Grafting in Patients of Different Ages|Clinical and Experimental Immunology|Oxford Academic. https://academic.oup.com/cei/article-abstract/114/1/26/6480346?redirectedFrom=fulltext.

[7] Pinheiro de Oliveira R., Hetzel M.P., dos Anjos Silva M., Dallegrave D., Friedman G. (2010). Mechanical ventilation with high tidal volume induces inflammation in patients without lung disease. Crit. Care.

[8] Clinical Features of Patients Infected with 2019 Novel Coronavirus in Wuhan, China—The Lancet. https://www.thelancet.com/journals/lancet/article/PIIS0140-6736(20)30183-5/fulltext.

[9] Volmering S., Block H., Boras M., Lowell C.A., Zarbock A. (2016). The Neutrophil Btk Signalosome Regulates Integrin Activation during Sterile Inflammation. Immunity.

[10] Retsky M., Demicheli R., Hrushesky W., Baum M., Gukas I. (2010). Surgery triggers outgrowth of latent distant disease in breast cancer: An inconvenient truth?. Cancers.

[11] Chamaraux-Tran T.-N., Piegeler T. (2017). The Amide Local Anesthetic Lidocaine in Cancer Surgery—Potential Antimetastatic Effects and Preservation of Immune Cell Function? A Narrative Review. Front. Med..

[12] Piegeler T., Votta-Velis E.G., Liu G., Place A.T., Schwartz D.E., Beck-Schimmer B., Minshall R.D., Borgeat A. (2012). Antimetastatic potential of amide-linked local anesthetics: Inhibition of lung adenocarcinoma cell migration and inflammatory Src signaling independent of sodium channel blockade. Anesthesiology.

[13] Chang Y.-C., Liu C.-L., Chen M.-J., Hsu Y.-W., Chen S.-N., Lin C.-H., Chen C.M., Yang F.M., Hu M.C. (2014). Local anesthetics induce apoptosis in human breast tumor cells. Anesth. Analg..

[14] Piegeler T., Schläpfer M., Dull R.O., Schwartz D.E., Borgeat A., Minshall R.D., Beck-Schimmer B. (2015). Clinically relevant concentrations of lidocaine and ropivacaine inhibit TNFα-induced invasion of lung adenocarcinoma cells in vitro by blocking the activation of Akt and focal adhesion kinase. Br. J. Anaesth..

[15] Chang Y.-C., Hsu Y.-C., Liu C.-L., Huang S.-Y., Hu M.-C., Cheng S.-P. (2014). Local Anesthetics Induce Apoptosis in Human Thyroid Cancer Cells through the Mitogen-Activated Protein Kinase Pathway. PLoS ONE.

[16] Le Gac G., Angenard G., Clément B., Laviolle B., Coulouarn C., Beloeil H. (2017). Local Anesthetics Inhibit the Growth of Human Hepatocellular Carcinoma Cells. Anesth. Analg..

[17] Xing W., Chen D.-T., Pan J.-H., Chen Y.-H., Yan Y., Li Q., Xue R.F., Yuan Y.F., Zeng W.A. (2017). Lidocaine Induces Apoptosis and Suppresses Tumor Growth in Human Hepatocellular Carcinoma Cells In Vitro and in a Xenograft Model In Vivo. Anesthesiology.

[18] Jaura A.I., Flood G., Gallagher H.C., Buggy D.J. (2014). Differential effects of serum from patients administered distinct anaesthetic techniques on apoptosis in breast cancer cells in vitro: A pilot study. Br. J. Anaesth..

[19] Schmidt W., Schmidt H., Bauer H., Gebhard M.M., Martin E. (1997). Influence of lidocaine on endotoxin-induced leukocyte-endothelial cell adhesion and macromolecular leakage in vivo. Anesthesiology.

[20] MacGregor R.R., Thorner R.E., Wright D.M. (1980). Lidocaine inhibits granulocyte adherence and prevents granulocyte delivery to inflammatory sites. Blood.

[21] Rancan L., Simón C., Marchal-Duval E., Casanova J., Paredes S.D., Calvo A., García C., Rincón D., Turrero A., Garutti I. (2016). Lidocaine Administration Controls MicroRNAs Alterations Observed After Lung Ischemia-Reperfusion Injury. Anesth. Analg..

[22] Jönsson A., Cassuto J., Tarnow P., Sinclair R., Bennett A., Tavares I.A. (1999). Effects of amide local anaesthetics on eicosanoid formation in burned skin. Acta Anaesthesiol. Scand..

[23] Flynn J.T. (1983). Effect of lidocaine on hepatic prostanoid production in vitro following 2,4-dinitrophenol administration. Adv. Shock Res..

[24] Modig J. (1989). Influence of regional anesthesia, local anesthetics, and sympathicomimetics on the pathophysiology of deep vein thrombosis. Acta Chir. Scand. Suppl..

[25] Lo B., Hönemann C.W., Kohrs R., Hollmann M.W., Polanowska-Grabowska R.K., Gear A.R., Durieux M.E. (2001). Local anesthetic actions on thromboxane-induced platelet aggregation. Anesth. Analg..

[26] Cassuto J., Tarnow P. (2003). Potent inhibition of burn pain without use of opiates. Burns. J. Int. Soc. Burn. Inj..

[27] Jönsson A., Cassuto J., Hanson B. (1991). Inhibition of burn pain by intravenous lignocaine infusion. Lancet Lond. Engl..

[28] Hendrickson H.S., van Dam-Mieras M.C. (1976). Local anesthetic inhibition of pancreatic phospholipase A2 action on lecithin monolayers. J. Lipid Res..

[29] Ma X., Yan W., He N. (2022). Lidocaine attenuates hypoxia/reoxygenation-induced inflammation, apoptosis and ferroptosis in lung epithelial cells by regulating the p38 MAPK pathway. Mol. Med. Rep..

[30] Cusati G., Garutti Martínez I., Vara Ameigeiras E. (2015). Universidad Complutense de Madrid Facultad de Medicina Departamento de Cirugía. Efecto de la Lidocaína en la Modulación del daño Pulmonar en un Modelo Experimental de Cirugía de Resección pulmonar en Cerdos. Ph.D. Thesis.

[31] Lin S., Jin P., Shao C., Lu W., Xiang Q., Jiang Z., Zhang Y., Bian J. (2020). Lidocaine attenuates lipopolysaccharide-induced inflammatory responses and protects against endotoxemia in mice by suppressing HIF1α-induced glycolysis. Int. Immunopharmacol..

[32] Zheng Y., Hou X., Yang S. (2019). Lidocaine Potentiates SOCS3 to Attenuate Inflammation in Microglia and Suppress Neuropathic Pain. Cell. Mol. Neurobiol..

[33] Zhang Y., Tao G.-J., Hu L., Qu J., Han Y., Zhang G., Qian Y., Jiang C.-Y., Liu W.-T. (2017). Lidocaine alleviates morphine tolerance via AMPK-SOCS3-dependent neuroinflammation suppression in the spinal cord. J. Neuroinflammation.

[34] Chiu K.M., Lu C.W., Lee M.Y., Wang M.J., Lin T.Y., Wang S.J. (2016). Neuroprotective and anti-inflammatory effects of lidocaine in kainic acid-injected rats. Neuroreport.

[35] Wang X.-X., Dai J., Wang Q., Deng H.-W., Liu Y., He G.-F., Guo H.-J., Li Y.-L. (2023). Intravenous lidocaine improves postoperative cognition in patients undergoing laparoscopic colorectal surgery: A randomized, double-blind, controlled study. BMC Anesthesiol..

[36] Beaussier M., Delbos A., Maurice-Szamburski A., Ecoffey C., Mercadal L. (2018). Perioperative Use of Intravenous Lidocaine. Drugs.

[37] Eipe N., Gupta S., Penning J. (2016). Intravenous lidocaine for acute pain: An evidence-based clinical update. BJA Educ..

[38] Lauretti G.R. (2008). Mechanisms of analgesia of intravenous lidocaine. Rev. Bras. Anestesiol..

[39] DeToledo J.C. (2000). Lidocaine and Seizures. Ther. Drug Monit..

[40] Yang S.Y., Kang H., Choi G.J., Shin H.Y., Baek C.W., Jung Y.H., Choi Y.S. (2014). Efficacy of intraperitoneal and intravenous lidocaine on pain relief after laparoscopic cholecystectomy. J. Int. Med. Res..

[41] Shakir F.T.Z., Sultan R., Siddiqui R., Shah M.Z., Javed A., Jamal A. (2023). Perioperative Intravenous Lidocaine Infusion for Postlaparoscopic Cholecystectomy Pain. J. Coll. Physicians Surg. Pak. JCPSP.

[42] Bakan M., Umutoglu T., Topuz U., Uysal H., Bayram M., Kadioglu H., Salihoglu Z. (2015). Opioid-free total intravenous anesthesia with propofol, dexmedetomidine and lidocaine infusions for laparoscopic cholecystectomy: A prospective, randomized, double-blinded study. Braz. J. Anesthesiol..

[43] Ortiz M.P., de Mello Godoy M.C., Schlosser R.S., Ortiz R.P., Godoy J.P.M., Santiago E.S., Rigo F.K., Beck V., Duarte T., Duarte M.M.M.F. (2016). Effect of endovenous lidocaine on analgesia and serum cytokines: Double-blinded and randomized trial. J. Clin. Anesth..

[44] Casas-Arroyave F.D., Osorno-Upegui S.C., Zamudio-Burbano M.A. (2023). Therapeutic efficacy of intravenous lidocaine infusion compared with thoracic epidural analgesia in major abdominal surgery: A noninferiority randomised clinical trial. Br. J. Anaesth..

[45] Swenson B.R., Gottschalk A., Wells L.T., Rowlingson J.C., Thompson P.W., Barclay M., Sawyer R.G., Friel C.M., Foley E., Durieux M.E. (2010). Intravenous lidocaine is as effective as epidural bupivacaine in reducing ileus duration, hospital stay, and pain after open colon resection: A randomized clinical trial. Reg. Anesth. Pain Med..

[46] Terkawi A.S., Tsang S., Kazemi A., Morton S., Luo R., Sanders D.T., Regali L.A., Columbano H., Kurtzeborn N.Y., Durieux M.E. (2016). A Clinical Comparison of Intravenous and Epidural Local Anesthetic for Major Abdominal Surgery. Reg. Anesth. Pain Med..

[47] Sun J., Wang S., Wang J., Gao X., Wang G. (2022). Effect of Intravenous Infusion of Lidocaine Compared with Ultrasound-Guided Transverse Abdominal Plane Block on the Quality of Postoperative Recovery in Patients Undergoing Laparoscopic Bariatric Surgery. Drug Des. Devel. Ther..

[48] Plass F., Nicolle C., Zamparini M., Al Issa G., Fiant A.L., Le Roux Y., Gérard J.L., Fischer M.O., Alvès A., Hanouz J.L. (2021). Effect of intra-operative intravenous lidocaine on opioid consumption after bariatric surgery: A prospective, randomised, blinded, placebo-controlled study. Anaesthesia.

[49] Vigneault L., Turgeon A.F., Côté D., Lauzier F., Zarychanski R., Moore L., McIntyre L.A., Nicole P.C., Fergusson D.A. (2011). Perioperative intravenous lidocaine infusion for postoperative pain control: A meta-analysis of randomized controlled trials. Can. J. Anaesth. J. Can. Anesth..

[50] De Oliveira G.S., Duncan K., Fitzgerald P., Nader A., Gould R.W., McCarthy R.J. (2014). Systemic lidocaine to improve quality of recovery after laparoscopic bariatric surgery: A randomized double-blinded placebo-controlled trial. Obes. Surg..

[51] Marret E., Rolin M., Beaussier M., Bonnet F. (2008). Meta-analysis of intravenous lidocaine and postoperative recovery after abdominal surgery. Br. J. Surg..

[52] Groudine S.B., Fisher H.A., Kaufman R.P., Patel M.K., Wilkins L.J., Mehta S.A., Lumb P.D. (1998). Intravenous lidocaine speeds the return of bowel function, decreases postoperative pain, and shortens hospital stay in patients undergoing radical retropubic prostatectomy. Anesth. Analg..

[53] Nakhli M.S., Kahloul M., Guizani T., Zedini C., Chaouch A., Naija W. (2018). Intravenous lidocaine as adjuvant to general anesthesia in renal surgery. Libyan J. Med..

[54] Wuethrich P.Y., Romero J., Burkhard F.C., Curatolo M. (2012). No benefit from perioperative intravenous lidocaine in laparoscopic renal surgery: A randomised, placebo-controlled study. Eur. J. Anaesthesiol..

[55] Grady M.V., Mascha E., Sessler D.I., Kurz A. (2012). The effect of perioperative intravenous lidocaine and ketamine on recovery after abdominal hysterectomy. Anesth. Analg..

[56] Bryson G.L., Charapov I., Krolczyk G., Taljaard M., Reid D. (2010). Intravenous lidocaine does not reduce length of hospital stay following abdominal hysterectomy. Can. J. Anaesth. J. Can. Anesth..

[57] El-Tahan M.R., Warda O.M., Diab D.G., Ramzy E.A., Matter M.K. (2009). A randomized study of the effects of perioperative i.v. lidocaine on hemodynamic and hormonal responses for cesarean section. J. Anesth..

[58] Terkawi A.S., Durieux M.E., Gottschalk A., Brenin D., Tiouririne M. (2014). Effect of intravenous lidocaine on postoperative recovery of patients undergoing mastectomy: A double-blind, placebo-controlled randomized trial. Reg. Anesth. Pain Med..

[59] Grigoras A., Lee P., Sattar F., Shorten G. (2012). Perioperative intravenous lidocaine decreases the incidence of persistent pain after breast surgery. Clin. J. Pain..

[60] Terkawi A.S., Sharma S., Durieux M.E., Thammishetti S., Brenin D., Tiouririne M. (2015). Perioperative lidocaine infusion reduces the incidence of post-mastectomy chronic pain: A double-blind, placebo-controlled randomized trial. Pain Physician.

[61] Klinger R.Y., Cooter M., Berger M., Podgoreanu M.V., Stafford-Smith M., Ortel T.L., Welsby I.J., Levy J.H., Rinder H.M., Newman M.F. (2016). Effect of intravenous lidocaine on the transcerebral inflammatory response during cardiac surgery: A randomized-controlled trial. Can. J. Anaesth. J. Can. Anesth..

[62] Cui W., Li Y., Li S., Wang R., Li J. (2010). Systemic administration of lidocaine reduces morphine requirements and postoperative pain of patients undergoing thoracic surgery after propofol-remifentanil-based anaesthesia. Eur. J. Anaesthesiol..

[63] Wang L., Sun J., Zhang X., Wang G. (2021). The Effect of Lidocaine on Postoperative Quality of Recovery and Lung Protection of Patients Undergoing Thoracoscopic Radical Resection of Lung Cancer. Drug Des. Devel. Ther..

[64] Yao Y., Jiang J., Lin W., Yu Y., Guo Y., Zheng X. (2021). Efficacy of systemic lidocaine on postoperative quality of recovery and analgesia after video-assisted thoracic surgery: A randomized controlled trial. J. Clin. Anesth..

[65] Martin F., Cherif K., Gentili M.E., Enel D., Abe E., Alvarez J.C., Mazoit J.X., Chauvin M., Bouhassira D., Fletcher D. (2008). Lack of impact of intravenous lidocaine on analgesia, functional recovery, and nociceptive pain threshold after total hip arthroplasty. Anesthesiology.

[66] Farag E., Ghobrial M., Sessler D.I., Dalton J.E., Liu J., Lee J.H., Zaky S., Benzel E., Bingaman W., Kurz A. (2013). Effect of perioperative intravenous lidocaine administration on pain, opioid consumption, and quality of life after complex spine surgery. Anesthesiology.

